# PP63 Perspectives Of Physical And Organic Disability Organizations On Health Technology Assessment Processes

**DOI:** 10.1017/S0266462324002344

**Published:** 2025-01-07

**Authors:** Laura Quintas Lorenzo, Isabel González Suárez, Patricia Gómez-Salgado, María José Faraldo Vallés

## Abstract

**Introduction:**

Effective participation of individuals with disabilities in health technology assessment (HTA) processes is paramount. Aware of the reality of people with physical and organic disabilities, COGAMI (a not-for-profit umbrella organization of disability associations) conducted an internal study to gather perspectives on the participation of people with disabilities in HTA processes.

**Methods:**

An ad hoc questionnaire of four open-ended questions was designed and distributed via email to COGAMI’s socio-health commission, representing 23 entities and 4,000 people in Galicia. A thematic analysis of the responses obtained was carried out.

**Results:**

Consensus underscores the fundamental role of individuals with disabilities and their representative organizations in HTA processes, though currently, only those with greater resources actively participate. The participants found that insufficient information reaching patient organizations hinders participation (e.g., lack of awareness in proposal submission), complicating their involvement. Additional challenges include accessibility and the digital divide. Proposed solutions involve enhancing communication channels and information accessibility, establishing collaborative frameworks nationally, and actively considering the disability condition to ensure a fair and equitable implementation.

**Conclusions:**

This study suggests the need for concrete actions to enhance the participation of individuals with disabilities in HTA processes. Recommendations include improving communication channels, capacity building, and recognizing disability as a key element in HTA.

